# Preparation and Characterization of Expanded Clay-Paraffin Wax-Geo-Polymer Composite Material

**DOI:** 10.3390/ma11112191

**Published:** 2018-11-06

**Authors:** Ahmed Hassan, Najif Ismail, Abdel-Hamid I. Mourad, Yasir Rashid, Mohammad S. Laghari

**Affiliations:** 1 College of Engineering, United Arab Emirates University, P.O. Box 15551, Al Ain 15551, UAE; ahmourad@uaeu.ac.ae (A.-H.I.M.); yasir.rashid@uaeu.ac.ae (Y.R.); mslaghari@uaeu.ac.ae (M.S.L.); 2 School of Engineering, Wellington Institute of Technology, Lower Hutt 5045, New Zealand; najif.ismail@weltec.ac.nz

**Keywords:** geo-polymer coating, phase change material, expanded clay, macro-encapsulation, vacuum impregnation

## Abstract

Paraffin-based phase change material (PCM) is impregnated into the pores of lightweight expanded clay aggregate (LECA) through vacuum impregnation to develop PCM containing macro-capsules of LECA. Three different grades of LECA varying in size and morphology are investigated to host the PCM to determine the impregnation effectiveness, viability for coating, and its stability. The produced LECA-PCM is coated with geopolymer paste (GP) to provide leak proofing during the phase change. The PCM is thermophysically characterized by employing differential scanning calorimetry (DSC) and the temperature history method (THM) to determine the phase transition and the latent heat. The stability of the macro-capsules is determined by weight loss through rapid thermal cycling (RTC) at elevated temperatures. Leakage of the PCM is tested using the diffusion-oozing circle test (DOCT). The results show that the GP coated LECA-PCM macro-capsules achieved 87 wt % impregnation efficiencies and no noticeable loss of PCM, which indicates leak proofing of the developed capsules up to 1000 RTC.

## 1. Introduction

Phase change materials (PCMs) have been extensively studied in different configurations in buildings in order to reduce heat gain in hot climates and to store and utilize thermal energy in cold climates [1]. A computational model reported improvement in thermal performance of the building by incorporating PCM into the gypsum wallboard. The technique is viable for new as well as retrofit buildings to reduce energy consumption and the capacity requirement of the HVAC system to maintain indoor thermal comfort [2]. Application of PCM in a layered wall with the different arrangement of layers of insulation material and air cavity is also investigated. The study reported 44% reduction in the heat transmission to indoors and peak temperature shifting of 2.6 h [3]. For tropical weather conditions, a thin layer of PCM with the cool colored building envelope is proposed. This study reported energy saving in the range of 5% to 12% during the year with relatively stable weather conditions [4]. PCM plaster has been investigated in a cold climate as an internal wall and ceiling finishing material in an experimental research. Using PCM plaster, indoor temperature stabilized at almost 20 °C even when outside temperature was below −5 °C. This assembly also helped in maintaining the indoor humidity [5].

Ramakrishnan et al. extended the use of PCM to cementitious materials to improve the thermal performance of buildings. Use of PCM in cement materials applied to the building envelope can reduce peak temperature by 4.43 °C [6]. A major issue facing the PCM inclusion into the building emerged to prevent the PCM from leakage within building fabric during the phase change. Micro-encapsulation has been extensively studied as a solution to PCM by containing PCM in a stable shell material, which protects the PCM against the influences of the environment and volume changes during the phase change [7]. A comprehensive analysis of the previous studies on encapsulating PCM in different shell material employing different methods are summarized in Table 1.

Although the reviewed encapsulant materials [8,9,10,11,12,13,14,15,16,17,18,19,20,21,22,23,24,25,26] have shown success in the encapsulation efficacy, the materials possess certain limitations as it relates to their building applications. Certain materials are toxic or expensive [26,27] and fragile when subjected to shear loads in buildings [24,28] or the encapsulation processes are energy intensive [15,29]. The Geo-polymer is considered a potential encapsulant since the material preparation involves least energy inputs being fabricated through wet chemistry. Additionally, the geopolymer concrete (GPC) undergoes a phase change from a liquid-powder mix to a solid state through a non-Newtonian fluid phase during its production processes. The non-Newtonian intermediate phase can be exploited to coat the GPC over a surface and to close the surface pores to prevent leakage through the surface. Jacob et al. used Geo-polymer coating to encapsulate molten salt eutectic to mitigate the problem of corrosion of shells’ material caging salts [30]. The research was conducted for thermal energy storage for high temperature applications up to 600 °C [31,32]. The current study aims to develop thermally enhanced lightweight composite materials for building applications. The objective is achieved by encapsulating PCM into a lightweight expanded clay aggregate. Although expanded clay offers higher PCM encapsulation efficacy due to larger pore density, its performance may deteriorate rapidly due to PCM leakage through pores during the phase change. The current study identifies Geo-polymer produced from industrial waste materials and dune sand (DS) through alkali-activated polymerization as a coating material to prevent leakage of the PCM encapsulated in expanded clay. The resultant composite is evaluated for the thermal storage capacity and the thermal cycling stability for several phase change cycles.

## 2. Materials and Methods

Industrial waste is used in the study by activating the pozzolanic effect with the strong alkali. Materials and their preparation methods are described below.

### 2.1. Materials

The materials are procured from the local market and are characterized to verify the contents and phases since they would eventually affect the GPC formation. However, the impact of the content and phases on the GPC formation is not studied in this research. Microstructure is characterized by using scanning electron microscopy (SEM, JCM-5000, JEOL Limited, Tokyo, Japan) to have insight to the raw material. Thermophysical properties of the PCM are verified using differential scanning calorimetry (DSC-AT Q200, TA Instruments, New Castle, DE, USA) and temperature history method (THM). Lastly, developed capsules are tested to determine their thermal cycling stability. The list of materials used in the experimental work are presented in Table 2.

**Figure 1:**
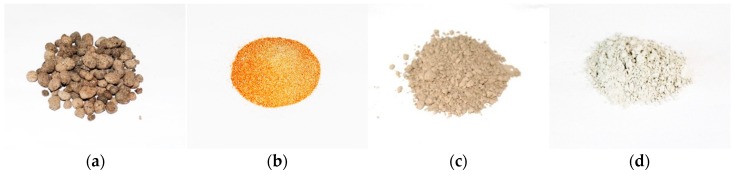
Photographs of (**a**) LECA, (**b**) DS, (**c**) FA, and (**d**) GGBS.

Ground granulated blast furnace slag (GGBS) and fly ash (FA) waste materials are investigated and found to form a GP by activating polymerization using a mixed solution of sodium silicate (Na_2_SiO_3_) and sodium hydroxide (NaOH). Based on the previous composition optimization [33], NaOH and Na_2_SiO_3_ were kept as 1:1.5 while the ratio of GGBS to FA to DS was fixed 1:3:6 in the geo-polymer paste composition. Three different grades of lightweight expanded clay aggregate (LECA) in terms of particle size and morphology were used. Figure 1 shows the photographs of the materials and the processes involved in the development of PCM capsules.

### 2.2. Materials Characterization

**Figure 2:**
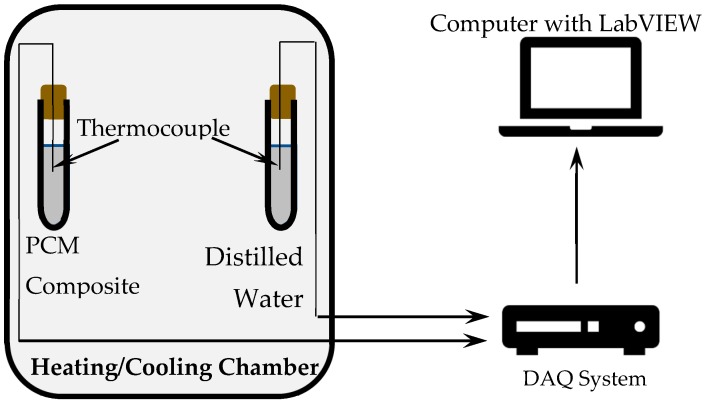
Schematic diagram of the experimental set-up used for the temperature history method.

Materials are characterized to test their composition, microstructure, and properties. FA, GGBS, and DS are characterized by X-ray powder diffraction (XRD, PW/1840, Philips, Amsterdam, The Netherlands), X-ray fluorescence (XRF, Lab Center XRF-1800, Shimadzu Corporation, Kyoto, Japan), and SEM. PCM was characterized to evaluate its melting on-set, melting peak, melting range, and heat of fusion through DSC and THM. The schematic diagram of the experimental set-up used for THM is shown in Figure 2. DSC can accommodate a very small sample size that make it vulnerable to the inaccuracies in the results [34,35]. The problem is more pronounced in case of heterogeneous materials [36], which undergo sub cooling. The THM solves the problems faced in DSC by accommodating larger sample sizes in the range of 20 to 50 g [37]. In the current experiment, THM employed an equal mass of distilled water and PCM contained in two identical 20 cm long glass tubes with the internal diameter of 1.8 cm and a wall thickness of 1 mm. These test tube dimensions assured the Biot number is below 0.1 in order to apply the lumped capacitance heat transfer model, according to Equation (1) [38].

(1) $$B_{i}=\frac{h_{c}\times L_{c}}{Κ}$$

where *h_c_* is the convective heat transfer co-efficient, *L_c_* is the characteristic length of the tube, and *Κ* is the effective thermal conductivity of the PCM and test tube material. With a system of such a small Biot number, heat transfer can be considered along the length of the wall surface when only recognizing it as the lumped capacitance method. Both test tubes were equipped with the k-type thermocouples (RS Components, Corby, UK). These thermocouples were fixed at the center point of the test tube by wrapping the wire of the thermocouple around a very thin but long insulated pin. The pin was fixed with the cork in the mouth of the tube to ensure the position of the thermocouple joint at the center. Both tubes were kept heated simultaneously at 45 °C in the heating chamber (ESPEC–Temperature and Humidity Chamber–Platninous J Series, Osaka, Japan) while the tubes were cooled by dipping into chilled water simultaneously. These thermocouples were attached to the data acquisition device (Compact DAQ (NI-cDAQ-9178), National Instruments, Austin, TX, USA) by using the module NI-9213, which was connected to the pc computer.

### 2.3. PCM Encapsulation

#### 2.3.1. Immersion

**Figure 3:**
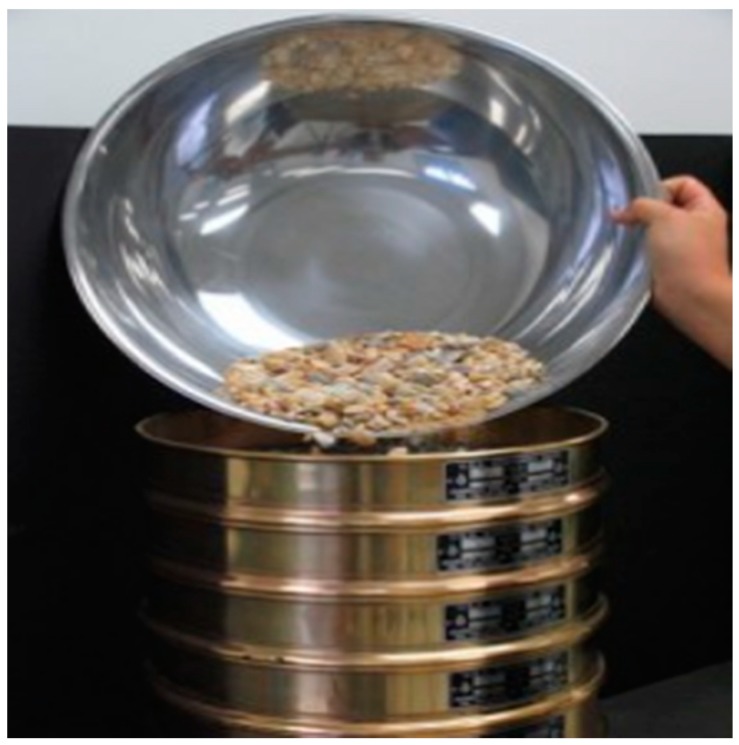
A representation of the immersion set-up.

A total of 100 g of each of LECA1, LECA2, and LECA3 were immersed separately in the liquid PCM, which is shown in Figure 3. The immersion time was varied from 30 min to 24 h while keeping the temperature of the PCM at 40 °C during the test. In another trial, the PCM temperature was changed from 35 °C to 70 °C to study the effect of PCM temperature on its penetration into LECA voids. The effect of stirring after 10 min of an interval was also tested. After immersion time, the left over molten PCM was drained into the metallic sieve. The prepared LECA-PCM was dried and weight was measured to determine the amount of PCM encapsulated inside the LECA.

#### 2.3.2. Vacuum Impregnation

Different trials of PCM impregnation were carried out to impregnate the maximum amount of PCM inside the pores of LECA. The procedure of the most effective trial is reported herein. LECA1, LECA2, and LECA3 were heated at 100 °C for 8 h to remove moisture content. The weighted amount (100 g) of the aggregates was added in the vacuum desiccator at −0.95 bar by employing a suction pump. The aggregate was spread in between two metallic sieves with a mesh size less than that of particles’ sizes to enhance surface contact between the aggregate and the PCM in a molten state. The melted PCM was injected from the top through a funnel to flow under gravity past the LECA through sieves. The PCM was kept injected inside the desiccator until the porous aggregate was fully submerged. The top sieve is installed to prevent LECA floating over the PCM surface when a vacuum is applied. The bottom sieve is installed to prevent settling of the heavier LECA-PCM mix when the vacuum is removed. The vacuum was applied for 30 min under PCM stirring once every 5 min where the vacuum desiccator was shaken to achieve the stirring. The PCM is assumed to keep impregnation into LECA as long as air bubbles keep appearing under vacuum in the melt desiccator containing LECA-PCM mix. The temperature of the mix is maintained substantially above the PCM melting point to prevent PCM solidification above the LECA surface during impregnation. The PCM temperature was maintained substantially higher than the solidification temperature to prevent PCM solidification within pores during impregnation to assure maximal encapsulation. 

#### 2.3.3. Coating

**Figure 4:**
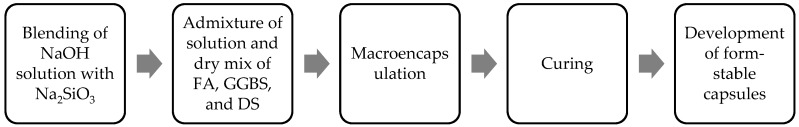
Illustration of the steps involved in encapsulation of PCM in LECA.

A total of 18 molar NaOH solutions were blended with the Na_2_SiO_3_ solution and shake well for five minutes to assure solution homogenization. The mixing ratios and solution molarities being applied in the current research were reported to achieve a maximum compressive strength [33]. Due to the exothermic nature of the reaction, the heated solution was kept for one day in ambient conditions to cool down prior to being poured into a dry mix of FA, GGBS, and DS. The FA, GGBS, and DS powders were mixed and homogenized in a steel container by using a mechanical stirrer simultaneously. Alkali solution was poured into the powder mix to develop GP paste. Macro capsules of LECA-PCM were added to the GP paste in the concrete mixer (Namson Trading GmbH, Frankfurt, Germany) to apply a GP layer around the LECA-PCM particles. As soon as the hardening of the GP layer started over the LECA-PCM surface, the macro capsules were taken out of the concrete mixer. The LECA-PCM capsules were rolled over a bench until complete hardening of the GP paste occurred. An illustration of the steps involved in the process are shown in Figure 4.

**Figure 5:**
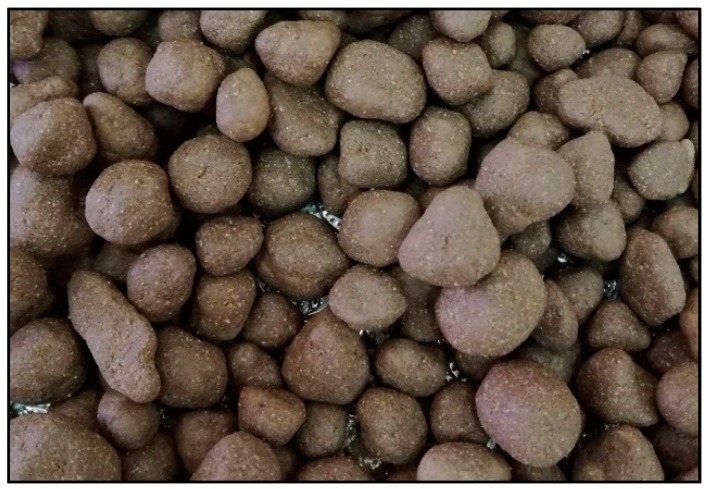
PCM impregnated LECA with geo-polymer coating.

The rolling of a spherical aggregate filled with PCM into the geo-polymer paste produced spherical capsules. The process yielded a GP-coated form stable LECA-PCM macro-capsules, which is shown in Figure 5. Newly produced capsules were kept at room temperature for 24 h for curing. 

### 2.4. Testing of Thermal Stability

#### 2.4.1. Weathering Test

GP-coated LECA-PCM capsules were kept outdoors for 60 days with temperatures fluctuating above and below the melting point of PCM to visually observe degradation of coating under solar radiation and temperature cycles. The ambient temperature fluctuated between 38 °C and 23 °C during the daytime and nighttime of the test duration.

#### 2.4.2. Controlled Indoor Test

**Figure 6:**
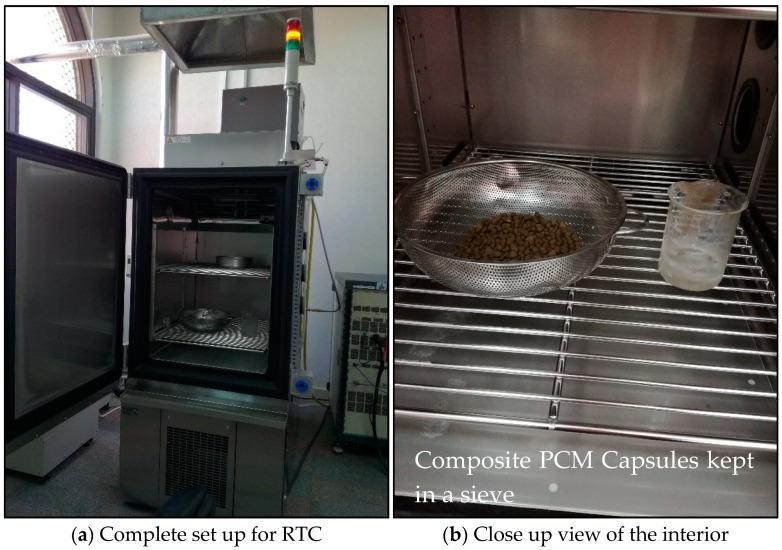
Set-up used for rapid thermal cycling: (**a**) complete view, and (**b**) a photo of the inside chamber.

The capsules were tested under RTC in a heating chamber (ESPEC–Temperature and Humidity Chamber–Platninous J Series, Osaka, Japan), which is shown in Figure 6. The temperature fluctuations during RTC were from 10 °C to 105 °C. The samples were kept in a perforated containment to let any produced vapors escape and this counted as weight loss. The samples were subjected to 1000 RTC and the weight was measured after 100 cycles along with visual observation to determine the apparent wetting of the surface.

#### 2.4.3. Diffusion-Oozing Circle Test

A DOCT was conducted to check the leakage of PCM out of GP-LECA-PCM capsules by following the method proposed in Reference [39]. In the test, a circle of 30 mm diameter was plotted on a filter paper and capsules of GP-LECA-PCM were placed inside the circle. It was heated up to 105 °C inside the heating chamber so that inside PCM can melt completely. After cooling it to a normal temperature, the capsules were removed and the circle was measured again. Leakage was measured by using Equation (2) [40].

(2) $$\eta =\frac{D_{LK}}{D_{SD}}\times 100\%$$

where the value of *η* will decide the leakage performance of the material, *D* is the diameter of the circle with the subscripts of *LK* representing the leakage circle and *SD* for the standard circle.

## 3. Results

Leak proofing of the GP-LECA-PCM capsules is the most important parameter of investigation. The results for impregnation efficiency, DSC, THM, RTC, and DOCT are presented below. 

### 3.1. Composition and Microstructure of the Materials

#### 3.1.1. X-ray Diffraction Analysis

**Figure 7:**
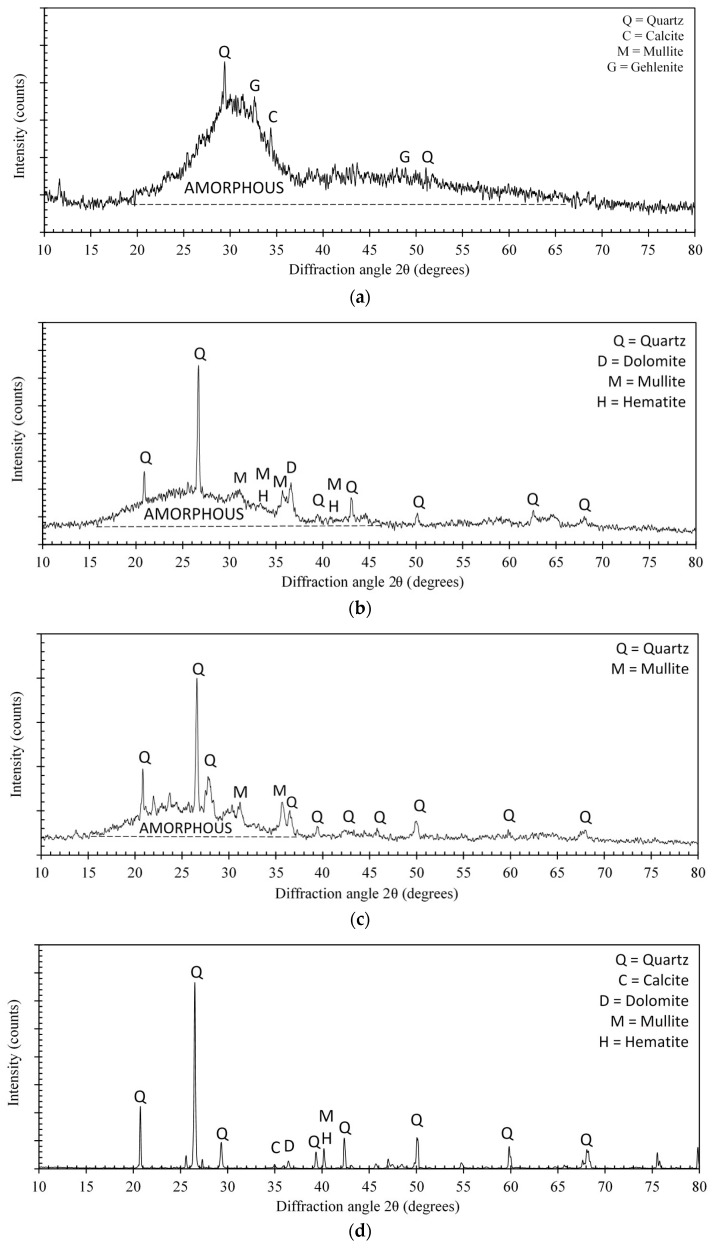
X-ray diffractograms of (**a**) GGBS; (**b**) FA; (**c**) LECA; and (**d**) DS.

The dry constituent materials were grounded to a fine powder and x-ray diffraction analysis was conducted with a Cu-Kα radiation at room temperature. The prepared samples were scanned at 2θ between 10° and 80°. The experimentally obtained X-ray diffractograms are shown in Figure 7.

The large halo located between 25° and 35° (2θ) in the X-ray diffractogram of GGBS (Figure 7a) indicates that it contains mostly amorphous compounds. The amorphous composition of GGBS is due to the quenching process where water is used during its production. Small reflections for Quartz (SiO_2_), Mullite (Al_6_Si_2_O_13_), and Gehlenite (Ca_2_Al(AlSiO_7_)) were also identified. Figure 7b displays the X-ray diffractogram of fly ash, which revealed several sharp crystalline peaks in 2θ range from 20° and 70°. The observed sharp crystalline peaks were assigned to the main crystalline phases of Quartz (SiO_2_), Mullite (Al_6_Si_2_O_13_), and Hematite (Fe_2_O_3_). The presence of these relatively inactive crystalline phases is typical in low-calcium flay ash. The wide diffusive hump shows the presence of a small quantity of amorphous solids as well. The ground LECA powder showed phase composition similar to that expected for autoclaved clays (Figure 7c). Quartz (SiO_2_) was the major crystalline phase identified in the dune sand (Figure 7d) with other minor phases identified to include Calcite (CaCO_3_), Dolomite (CaMg(CO_3_)_2_), Mullite (3Al_2_O_32_SiO_2_), and Hematite (α-Fe_2_O_3_).

#### 3.1.2. Scanning Electron Microscopy Analysis

**Figure 8:**
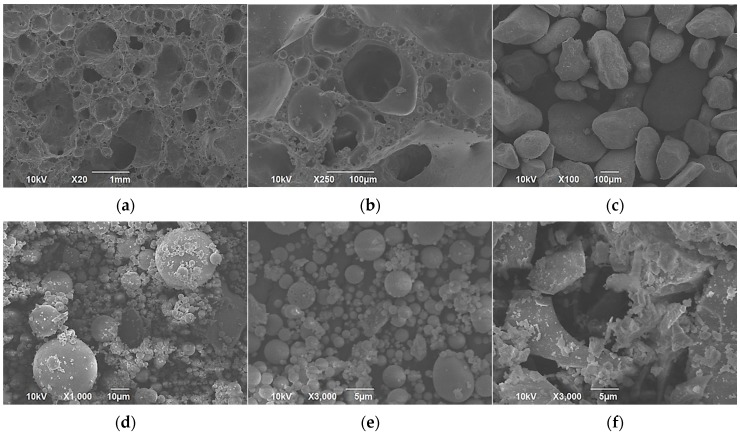
Microstructure of (**a**) LECA Mag. 20, (**b**) LECA Mag. 250, (**c**) DS Mag. 100, (**d**) FA Mag. 1000, (**e**) FA Mag. 3000, and (**f**) GGBS Mag. 3000.

The scanning electron microscopic (SEM) analysis was carried out by using accelerating voltages of 10 kV and 15 kV. Interconnected porosity of LECA can be observed in Figure 8a,b. The pore size was not uniform and ranged from a few μm to almost 1 mm. This porosity can be installed in buildings for thermal insulation, sound proofing, and lightness of concrete. This research exploited its ability to host PCM in its porosity because of its interconnected type. A microstructure of DS was noted to be nodular with the size range approximately 80 μm to 200 μm (Figure 8c). SEM investigation of FA revealed that its particles were spherical in shape and mainly smaller than 30 μm (Figure 8d) but had a broad particle size distribution (Figure 8e). GGBS particles were noted to have different microstructures (Figure 8f) i.e., coarser than fly ash and were angular in structure with a mean particle size of about 27 μm. 

**Figure 9:**
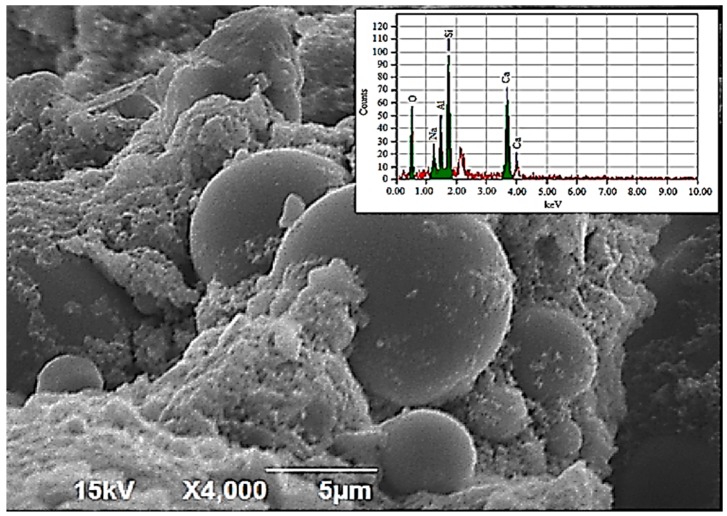
Micrograph and EDX plot for the pulverized geo-polymer paste.

Detailed SEM and EDX analysis of the geo-polymer paste has been reported by authors in a precedent study [33] and key findings are reported here for relevance. Figure 9 shows the micrograph of the geo-polymer. It can be observed that FA spheres were intermixed with angular slag particles and reaction products can adhere to the surface of FA spheres. To further characterize the reaction products, energy dispersive X-ray (EDX) spot analysis was employed. The EDX plot highlighted the presence of calcium (Ca), silicon (Si), sodium (Na), aluminum (Al), and oxygen (O). This indicated that an aluminium-modified C–S–H gel co-existed with an N–A–S–H geopolymer gel. A silicon-to-aluminum (Si/Al) ratio of 1.80 was reported in the aluminosilicate phase.

#### 3.1.3. X-ray Fluorescence Analysis

Received densities and chemical composition of the GGBS, FA, and DS are presented in Table 3. The FA used here was categorized as class F in accordance with ASTM C618 [41].

### 3.2. Thermo-Physical Properties of PCM

#### 3.2.1. Differential Scanning Calorimetry

**Figure 10:**
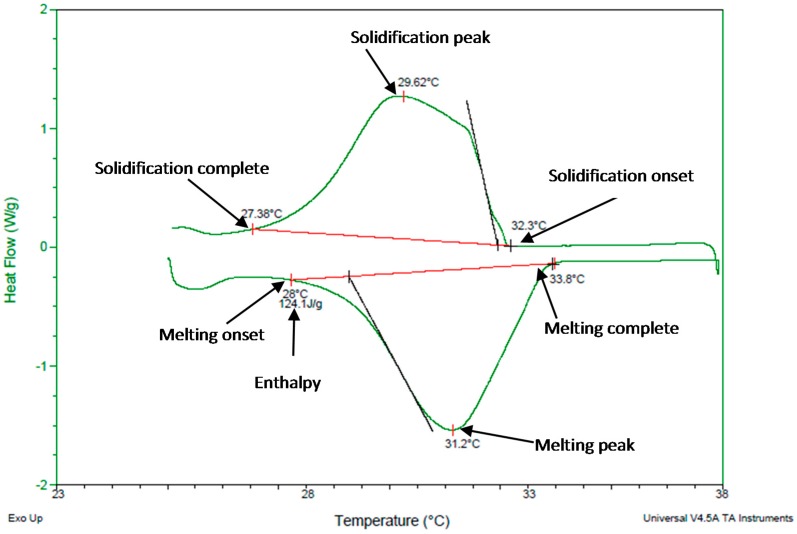
Thermogram of PCM using DSC.

Figure 10 shows the DSC thermogram of a 5-mg PCM sample at a scanning rate of 1 °C/min. The heating run shows that heat absorption starts at 28 °C and is completed at 33.8 °C with the peak at 31.2 °C with a heat of fusion of 124.1 J/g. The cooling run shows that solidification started at 32.3 °C and completed at 27.38 °C with the peak at 29.62 °C. DSC results agree with the manufacturer’s catalogue melting points of 27–33 °C while it substantially disagrees with the solidification range of 33–27 °C [42].

#### 3.2.2. Temperature History Method

**Figure 11:**
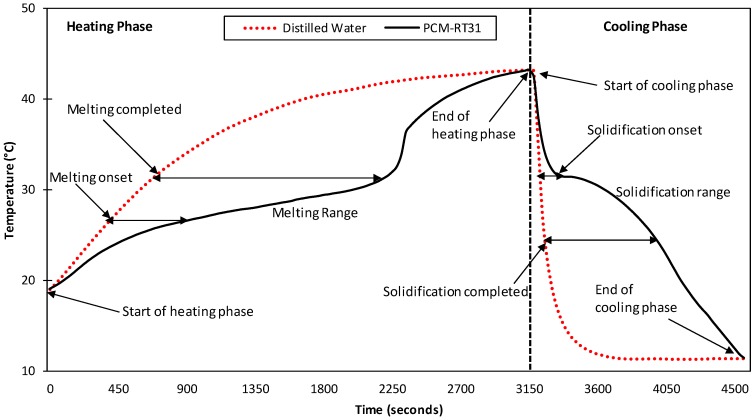
Temperature History Method (THM) curve for the PCM.

The THM results slightly disagrees with the DSC in melting and solidification temperatures possibly due to the hysteresis nature of the material and thermal gradient inside the PCM sample [34] or the uncertainty in the measuring instruments [43]. In THM, the end of melting in the heating phase and solidification onset in the cooling phase were almost the same at 32 °C as shown in Figure 11. During heating, it represents a delay in the PCM temperature rise when compared to distilled water due to its higher heat of fusion. The melting initiated at 27 °C and completed at 32 °C was represented by a drop in the temperature gradient, which indicates latent heat absorbed by the PCM. In comparison, the temperature rise for water is consistent and uniform. The difference in the gradient of the temperature rise of the materials is also visible in the cooling regime. It represents a delay in the cooling of PCM as compared to water due to higher heat release of the PCM during its phase transition. For the cooling phase, solidification started at 33 °C and was completed at 26.5 °C. The heating phase was completed in almost 3150 seconds while cooling required 1350 s. The difference in time required is due to different heating and cooling rates in both phases. Quenching of both tubes in cold water at 10 °C enhanced the cooling rate, which resulted in rapid cooling and a sharp curve when compared to the heating phase.

### 3.3. Impregnation Efficiency

#### 3.3.1. Immersion

There was no observable difference in weight gain of the aggregate and consequently no impregnation. Therefore, the immersion technique is not effective and should not be used.

#### 3.3.2. Vacuum Impregnation

The size of the LECA particle and its morphology have a marginal effect on its absorption efficiency. The crushing of the particles of LECA2 and LECA3 slightly increased the PCM absorption from 83 wt % to 87 wt %. This increment may be attributed to the utmost filling of pores because the surface treatment of LECA was removed when crushed. For further investigations, crushed particles and LECA3 were not considered because they could not be rolled in the GP paste due to irregular morphology. In the best case, a maximum of 87 wt % was achieved in LECA1 but a smaller size of the LECA caused agglomeration while coating with GP. Therefore, LECA2 were finalized for further research because of comparable absorption of 83 wt %, which is a suitable size and regular shape for GP coating. In a study, only 31 wt % was impregnated into LECA using vacuum impregnation [44,45]. Yet, Na_2_SiO_3_ was impregnated with the density at much higher levels than that of PCM. Absorption efficiency is important for a compact but dense thermal energy storage system. The higher the quantity, PCM is filled in the pores of the same LECA as compared to the smaller quantity required when used in the building components for thermal energy management. Therefore, by developing denser particles and using less quantity, it will generate the same thermal effects and less compromised structural strength.

### 3.4. Thermal Stability

#### 3.4.1. Weather and Rapid Thermal Cycling

**Figure 12:**
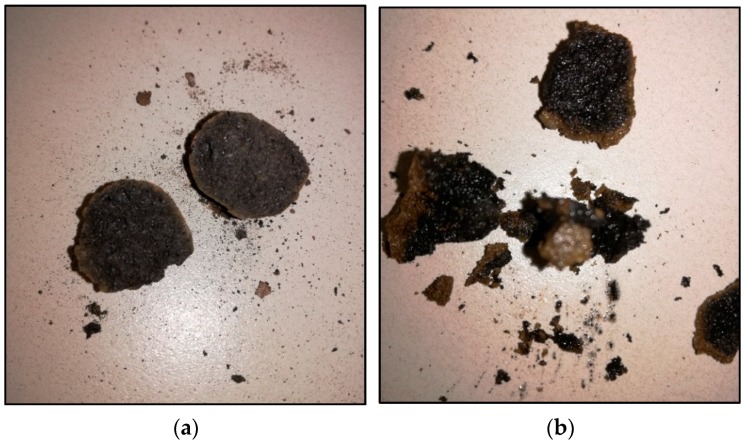
Fracture surfaces of (**a**) LECA and (**b**) GP-LECA-PCM.

Thermal stresses may induce cracks or rupture on the coating layer. No apparent sign of leakage or rupture was observed over the GP coated LECA-PCM surface indicating the durability of the coating. To validate the presence of PCM inside the GP-LECA-PCM, a capsule at the high temperature was crushed with a high impact load. A good amount of PCM was observed inside the GP shell in a molten form, which is shown in Figure 12. This confirms the longevity of the coating material. Two different types of expanded clay aggregates have been investigated for PCM absorption to develop thermal energy storage concrete. Although the reported value for the PCM absorption is 89.8% at the maximum, the investigation included only the weight loss method to test the thermal stability in a narrow range of thermal cycles between 10 °C to 60 °C [46]. The study has limitations because, at an even higher temperature, materials will behave differently because of the mismatch of volume changes of PCM, the aggregate, and its coating. Aguayo et al. used four different types of LECA to host PCM and achieved 21.2% PCM absorption efficiency at the maximum. The study investigated cement-based insulation to reduce the insulation layer thickness with the dampened temperature peaks [47].

#### 3.4.2. Diffusion-Oozing Circle Test

**Figure 13:**
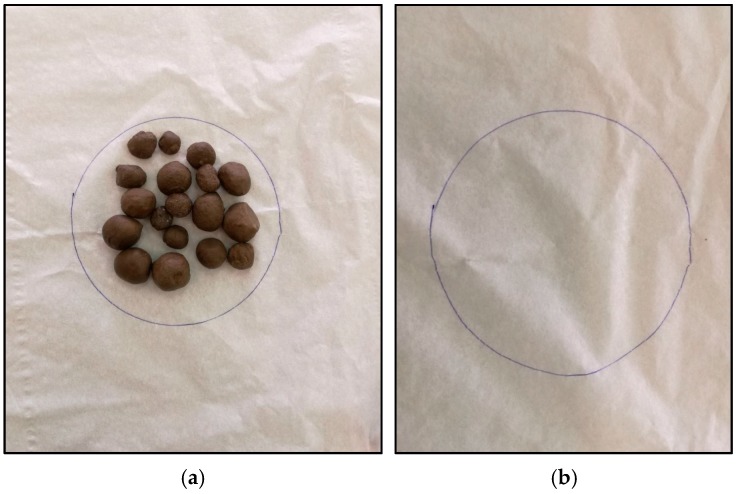
Exudation stability of GP-LECA-PCM. (**a**) Filter paper with GP-LECA-PCM; (**b**) Filter paper after removing GP-LECA-PCM.

The test claims that the maximum of 15% increase in the diameter of the circle is acceptable for leakage of PCM out of the porous media [48]. Results of our study revealed there was absolutely no increase in the ooze circle, which indicated the perfect leak proofing of the capsules. Figure 13 shows the filter paper with and without GP-LECA-PCM capsules on its surface after exposing it to higher temperatures. Hence, the materials and methods can be used for leak proofing of PCMs contained in the porous media.

## 4. Conclusions

A paraffin based PCM is characterized through DSC and THM for building integration aimed at enhancing the thermal performance of buildings. The material exhibited a reasonable thermal energy storage capacity of 124.1 J/g with a phase transition temperature of 28 °C being close to a human thermal comfort. The PCM is macro-encapsulated into porous lightweight expanded clay aggregate (LECA) by vacuum impregnation to form LECA-PCM composite capsules. A low porosity Geo-polymer paste is prepared through alkali-activated polymerization of fly ash, glass slag, and dune sand and applied to LECA-PCM surface by mechanically rolling capsules over the paste. The paste forms a uniform layer that hardens over the LECA-PCM surface and fills the open pores, which forms a leak proof layer that prevents PCM leakage during the liquid phase. Thermal cycling durability of the leak proof layer is tested both under an indoor temperature change of 15 °C to 105 °C and outdoor temperatures irradiated by solar radiation undergoing a temperature change of 23 °C to 65 °C. The protective coating remained intact for the tested duration showing no obvious surface wetting as well as weight loss. It is, therefore, concluded that the Geo-polymer coating is effective at preventing the leakage of macro-encapsulated PCMs contained in a porous shell under extreme indoors and outdoors conditions for limited thermal cycles. The durability of the leak proof layer still needs to tested for longer exposure times in a range of several years through rapid thermal cycling indoors and outdoors. 

## Figures and Tables

**Table 1:** Summary of findings of experimental research on PCM encapsulation using different core and shell materials, production processes, and reported findings.

Production Process	Core Material	Shell Material	Findings
Sol–gel method [8]	Polyethylene glycol	Silicon dioxide	*H_m_* was in the range of 102.8–111.1 J/g. There was no change on enthalpy and transition temperature after 50 thermal cycles. Polyethylene glycol decomposes at 410 °C.
Emulsion polymerization [9]	Paraffin and palmitic acid	Styrene and ethyl acrylate	*η* was successful with 32.7 wt % of paraffin and 47.8 wt % palmitic acid. Φ was 165 nm and 265 nm for both *CMs* and they don’t decompose up to 200 °C.
Emulsion polymerization [10]	Caprylic (octanoic) acid	Polystyrene	Crosslinking agent had a direct impact on the encapsulation efficiency. Efficiency was compromised with repeatability.
Miniemulsion polymerization [11]	n-alkanes	Polystyrene	Thermal stability after RTC was reported. *M_p_* range was from 20 °C to 35.9 °C and *H_m_* range was 61.2 J/g to 46.1 J/g.
Mini-emulsion polymerization [7]	Hexadecane	Urea–formaldehyde resin	The results indicated that the nano-capsules have a smooth surface and Φ was 270 nm. Capsules were stable when heated at 100 °C for 72 h after encapsulation decreased the undercooling of hexadecane by 94%.
Mini-emulsion polymerization [12]	n-octadecane	Poly (ethyl methacrylate) + poly (methyl methacrylate)	Φ was 119 nm, *M_p_* and *H_m_* were 32.7 °C and 198.5 J/g respectively. The capsules have *η* of 89.5%.
In situ polymerization [13]	Butyl stearate and paraffin	Poly (methyl methacrylate-co-divinylbenzene)	Φ was 5–10 μm. Capsules decomposes at temperatures above 200 °C. Capsules were thermally stable after 50 cycles.
In situ polymerization [14]	Dodecanol	High-density polyethylene	*η* was successful yielding different sizes of the capsules in the range of 0.83 μm to 14.4 μm. Excellent thermal storage ability was good thermal stability reported.
Emulsion-solvent evaporation [15]	n-hexadecane	Ethyl cellulose	*M_p_* ranges from 18.5 °C to 19.5 °C while *H_m_* ranges from 137.8 J/g to 147.1 J/g. The shell had porosity and leakage was observed.
Solvent extraction [16]	Sodium nitrate	Perhydropolysilazane	*SM* in the capsules was 85 wt % while Φ was non-uniform with the range of 0.4 μm to 140 μm. *SM* was very stable at high temperatures of 350 °C.
Suspension-like polymerization [17]	n-octadecane	Poly (stearyl methacrylate)	Particles have a spherical profile with an average φ of 5 μm and 21 μm. Good thermal energy storage and thermal regulation potential was reported.
Suspension-like polymerization [18]	Paraffin	Poly Methyl Methacrylate	89.5 wt % of *CM* was encapsulated successfully with good thermal stability. Nano particles of 0.1 μm to 19 μm and micro particles of 94 μm were produced.
Suspension-like polymerization [19]	n-octadecane	Polymethylmethacrylate	Microcapsules have a high thermal storage capability, enhanced thermal reliability and stability, and increased thermal conductivity.
Crosslinking and blending [20]	Paraffin	Cross-linking structure	74 wt % of the *CM* was contained in the matrix successfully with the *H_m_* of 210.6 J/g. The samples were observed to be dry when heated up to 100 °C.
Vacuum Impregnation [21]	Polyethylene glycol	Diatomite	*M_p_* of the composite PCM was 27.7 °C and *H_m_* of 87.09 J/g. An addition of expanded graphite increased the thermal conductivity of the composite.
Vacuum impregnation [22]	Polyethylene glycol	Diatomite/carbon nanotubes	No leakage of PCM was observed. *M_p_* of the composite was 8 °C with *H_m_* of 62.9 J/g.
Vacuum impregnation [23]	Capric acid-myristic acid	Cement	Composite’s *M_p_* and *H_m_* were 21.13 °C and 41.78 J/g, respectively. A temperature difference of 0.78 °C in the indoor space was measured by using this composite.
Vacuum impregnation [24]	Capric acid-palmitic acid	Silica fume, carbon nano tube	*M_p_* range of different compositions was 19 to 26 °C and *H_m_* was 46 to 49 J/g. Good thermal and chemical stability was reported after 1000 cycles.
Fluidized bed method [25]	Bischofite	Acrylic	Encapsulation efficiency of up to 95% was achieved. Microcapsules had excellent *M_p_* and *H_m_* compared to the original PCM.
Melt coaxial electrospray [26]	n-octadecane	Sodium alginate	56 wt % of paraffin was contained in the microcapsules with *Φ* less than 100 μm. This technique offers good results regarding the encapsulation of PCMs.

*η*—Microencapsulation, *Φ*—Particle size, *H_m_*—Latent heat of fusion, *M_p_*—Melting point, *SM*—Shell material, and *CM*—Core material.

**Table 2:** List of materials used in the experiments.

Materials	Density	Particle Size
Lightweight expanded clay aggregate (LECA1)	421 kg/m^3^	1–4 mm
Lightweight expanded clay aggregate (LECA2)	369 kg/m^3^	4–10 mm
Lightweight expanded clay aggregate (LECA3)	340 kg/m^3^	4–10 mm
Paraffin-based phase change material	0.88 kg/L for solid 0.76 kg/L for liquid	Liquid/solid
Sodium Hydroxide (NaOH)	1.19 kg/L	Liquid
Sodium silicate (Na_2_SiO_3_)	1.39 kg/L	Liquid
Ground granulated blast furnace slag (GGBS)	1236 kg/m^3^	0.2–70 µm
Fly Ash (FA)	1262 kg/m^3^	3–70 µm
Dune Sand (DS)	1693 kg/m^3^	80–500 µm

**Table 3:** X-ray fluorescence (XRF) test results.

Constituent	SiO_2_ %	Al_2_O_3_ %	Fe_2_O_3_ %	CaO %	MgO %	LOI %
Fly ash	48	23	12.5	3.2	1.5	1.1
Slag	34.7	14.4	0.8	41.9	6.8	1.1
Dune sand	64.9	3	0.7	14.1	1.3	0.5
